# The Throat and Nose, and Their Diseases

**Published:** 1891-03

**Authors:** 


*The Throat and Nose, and their Diseases.* By LENNOX BROWNE, F.R.C.S. Ed. Third Edition. London: BAILLIÈRE, TINDALL & COX. 1890.

*Diseases of the Nose and its Accessory Cavities.* By W. SPENCER WATSON, F.R.C.S. Eng., B.M. Lond. Second Edition. London: H. K. LEWIS. 1890.

*A Handbook of Diseases of the Nose and Naso-pharynx.* By JAMES B. BALL, M.D. Lond., M.R.C.P. London: H. K. LEWIS. 1890.

Of these three books which deal with disease of the throat and nose, the first one no longer needs any introduction. We notice, however, that its author has changed the title of his work, and the additional pages on rhinology render the book distinctly more valuable than the previous editions. Mr. Lennox Browne evidently does not believe in specialising too narrowly, because he considers that there is an intimate relationship between disease of the so-called erectile tissue in the nasal turbinated bodies and that of the generative organs of both sexes, so that the gynæcologist and rhinologist are more nearly allied in their work than we are accustomed to suspect. We do not, however, agree with him that it is in accordance with common experience that atrophic rhinitis is closely connected with wasting or non-development of the female generative organs. We think that he might with advantage elaborate what he has written on ear disease, because the majority of ailments of that organ are secondary to affections of the nose or naso-pharynx.

The chapter on deviations and deformities of the septum is admirably illustrated by drawings, which show at a glance the operative measures necessary for the removal of the pathological condition. We are glad to find that Woakes' mischievous ideas as to the frequency of the occurrence of "necrosing ethmoiditis" are condemned. The chapters on diphtheria, lupus, and leprosy are exceedingly good; but we doubt if the theory that leprosy is an extremely attenuated tuberculosis as regards its progress and duration, but more virulent in its contagiousness and in the extent of its ravages, can be accepted.

The book shows abundant evidence of the author's skill as an artist, is well printed and bound, and is worthy to take its place on our shelves as a standard volume on the diseases with which it deals.

Mr. Spencer Watson's book thoroughly covers the whole ground of rhinology; and, besides this, contains articles by Dr. Robert Liveing, Mr. William Adams, and Mr. Cumberbatch.

A perfect system of classification and a methodical description of disease of the nose and accessory cavities has only recently been attempted; and we consider that Mr. Watson, by dividing his sections into subsections, and so gradually working out distinct pathological conditions, aids us in our study of the organ, and helps us to a right treatment of the numerous obstacles to the performance of the respiratory or olfactory function, such as deformities of the septum, spurs, polypi, and hypertrophic rhinitis, all of which frequently coexist in the nostril, and must be accurately dealt with if we are to effect a cure.

Numerous authors are referred to, many illustrative cases quoted, and the book is very well illustrated, and goes further than some other works of a similar character, Rhinoplastic operations, diseases of the skin and subcutaneous tissue of the nose, and intra-cranial complications of nasal affections being dealt with.

Dr. Ball's book is a handy *resumé* of the subject; but it appears at a time when several more elaborate works on the nose have just been published, which will militate against its being read.

